# Knowledge, motivation, self-efficacy, and their association with insecticidal net use among pregnant women in a secondary health centre in Maiduguri, Nigeria

**DOI:** 10.1186/s12936-018-2518-8

**Published:** 2018-10-12

**Authors:** Ahmed Dahiru Balami, Salmiah Md Said, Nor Afiah Mohd Zulkefli, Bachok Norsa’adah, Bala Audu

**Affiliations:** 1 Department of Community Health, Faculty of Medicine and Health Sciences, Universiti Putra Malaysia, Selangor Darul Ehsan, Malaysia; 2 Unit of Biostatistics and Research Methodology, Universiti Sains Malaysia, Kota Bharu, Malaysia; 3 Department of Obstetrics and Gynaecology, University of Maiduguri, Borno State, Nigeria

**Keywords:** Knowledge, Motivation, Self-efficacy, Insecticide treated net, Pregnant women, Nigeria

## Abstract

**Background:**

Despite the high prevalence of malaria among pregnant women and its associated complications, the level of compliance with insecticide-treated nets (ITN) remains very low. Motivation and self-efficacy have been reported as important determinants of health behaviour, and may be important factors to consider in developing health intervention programmes. The aim of this study was to determine the knowledge, motivation and self-efficacy of ITN use, and their association with its practice, among pregnant women in a secondary health centre in Maiduguri.

**Methods:**

The study utilized a cross-sectional study design, using a structured and pre-tested questionnaire to obtain information from 380 respondents. Respondents were classified as ITN users if they slept under an ITN for at least 3 days in a week, while those who did not at all, or slept under it less frequently were classified as ITN non-users. Chi squared test was performed to test the bivariate association between ITN use and each of the items of the questionnaire. A further multivariate logistic regression was performed to determine the predictors of ITN use.

**Results:**

The respondents’ ages ranged from 15 to 45 years, with median (interquartile range) age of 25 (8) years. Eighty percent of them were aware of ITN, but 50.5% believed ITNs could be dangerous. Only 5.5% and 0.8% respectively felt that sleeping under and ITN was either just bad or very bad for their health. Thirty-five percent of the respondents were ITN users. Not having a previous miscarriage (OR = 2.38; 95% CI 1.41–4.03, *p *= 0.001), knowledge that ITNs were not to be washed after every 1 month (OR = 3.60; 95% CI 1.18–11.06), significant others thinking they should sleep under an ITN (OR = 3.06; 95% CI 1.35–6.96), ability to effectively persuade others to sleep under an ITN (OR = 2.37; 95% CI 1.14–4.94) were significantly associated with ITN use.

**Conclusions:**

A large proportion of pregnant women in this study were not sleeping under ITNs. The development of health promotion interventions aimed at boosting their self-efficacies for ITN use, and improving social support from their spouses are, therefore, recommended. Health education on ITN use should also be incorporated into post-abortal management.

## Background

Malaria remains a serious public health problem among pregnant women in Nigeria, as high prevalence has been recorded even among antenatal care attendees in different parts of the country [1–5]. Similarly, through the years 2009–2015, high prevalence, ranging from 33.9 to 60.3% has been reported in the city of Maiduguri, Nigeria [6–10]. Malaria is associated with several complications, including abortions [11], anaemia [12–15], pre-term delivery [16, 17], stillbirth [18, 19] and low birth weight [20, 21].

The World Health Organization (WHO) recommends the use of insecticide-treated nets (ITN), intermittent preventive treatment with sulfadoxine–pyrimethamine (IPT–SP) and prompt treatment of malaria and anaemia, for all pregnant women in sub-Saharan Africa [22]. A systematic review of five trials, of which four were conducted in Africa, and the other in Thailand, had shown that ITN use was effective in reducing the incidence of placental parasitaemia, low birth weight and miscarriage/stillbirth [23]. Results from another trial had also shown that a judicious use of ITNs by pregnant women in malaria endemic areas was very effective in reducing the incidence of both malaria infection and its complications to an extent where taking IPT–SP did not even provide additional benefits [24]. Despite these recommendations by the WHO [22], the practice of sleeping under an ITN has been generally poor. The Nigerian National Demographic and Health Survey of 2008 reports that out of 34,070 households sampled, only 8.0% owned at least one ITN, and only 44.4% of the women living in households with an ITN were sleeping under them [25]. In the subsequent survey of 2013, the proportions of pregnant women who slept under any type of net the night before the survey were 17.4% and 18.4% for rural and urban dwellers respectively, while only 13.8% of the pregnant women in Borno state reported sleeping under an ITN the night before the survey [26]. In a tertiary health centre in Maiduguri, Borno state, only 2.3% of antenatal care attendees were sleeping under an ITN [7].

Motivation has been identified as an important factor in the performance of health behaviours, even among persons with adequate knowledge of the particular health behaviour [27]. Motivation comprises of personal motivation and social motivation [28]. Personal motivation entails the beliefs about the consequences of performing a particular task, while social motivation entails perception of the possible level of support from significant others to perform those tasks [29] The role of social motivation is also buttressed by findings of a systematic review which identified household decision as an important predictor of ITN use among pregnant women in Africa [30]. Self-efficacy on the other hand refers to a person’s perception of his/her abilities to perform a certain task [31]. It is believed to play a great role in determining how much a person is able to cope with, and sustain efforts to perform a task, in the face of obstacles [32]. A study among women in the Democratic Republic of Congo revealed that higher self-efficacy for ITN use was a predictor of its use [33]. Those who were confident of being able to hang or use an ITN were also more likely to sleep under it, compared to those who were not [34].

As previous studies have pointed out the important roles knowledge [35, 36], motivation [37] and self-efficacy [33, 34] play in influencing health behaviour, identifying the association between ITN use and these factors, would improve the present understanding of ITN use. It would also guide the development of more focused interventions to promote these practices, since individual items of these constructs would be studied. The aim of this study was to determine the level of knowledge, motivation, self-efficacy, and their association with ITN use among pregnant women at the State Specialist Hospital, Maiduguri, Nigeria.

## Methods

### Study area

The study area was Maiduguri, the Borno state capital in north-eastern Nigeria. It has a population of 540,016, comprising of 282,409 males and 257,607 females [38], and a literacy rate of 30.2% for English language, and 33.1% for any language among its adult females [39]. The study location was the ante-natal care clinic of the State Specialist Hospital, Maiduguri. This location was chosen because it is the biggest of the three state hospitals in Maiduguri; centrally located in the city; geographically most accessible of all the three hospitals, and has the highest patient load. Also in a previous study, less than half of the women attending the clinic reported sleeping under an ITN [10].

### Study design and study subjects

A cross-sectional study design was used for this study, recruiting participants at their first ante-natal care visits. The study was to be extended to a prospective intervention study which had pregnancy outcomes as part of its dependent variables, and as such, those who were not resident in Maiduguri, and those with hypertension, and/or diabetes mellitus were excluded, as these conditions could affect the pregnancy outcomes [40–42]. The one proportion formula was used to calculate the minimum required sample size [43]. The expected proportion (P) was substituted with the prevalence of ITN use among pregnant women in Borno State [26], while the Z-statistic and precision level (d) were substituted with 1.96 and 0.05 respectively [44], to obtain a minimum sample size requirement of 183 respondents. Respondents were recruited in batches, from eight consecutive antenatal booking clinic sessions, with each clinic session having a total of approximately 150 antenatal care attendees, who sat on the ten rowed seats in the waiting area. A systematic random sampling method was used to select respondents from the eligible attendees, based on the row and position in which they were seated, in the waiting area. This was done by serially going through the rows in which they were seated, starting from the front row, selecting one eligible attendee, and skipping the next eligible person to select other one, up to the last eligible person seated on the last row.

### Study instrument and data collection

The questionnaire used for this study was developed based on the information-motivation-behavioural skills (IMB) model, and consisted of five sections (respondents’ characteristics, knowledge of ITN, motivation, self-efficacy and practice of ITN use) (Appendix). Section one asked questions on respondents’ socio-demographic characteristics as well as some obstetric and gynaecological factors. Items of the second section, which was on knowledge of ITN, were derived from questionnaires of previous researchers [45–47] after obtaining their consent. This section had a total of 12 questions, each with three options, ‘Yes’, ‘No’ and ‘I don’t know’, which were re-coded as ‘Correct’, ‘Incorrect’ or ‘I don’t know’. The third and fourth sections were, respectively, on motivation and self-efficacy for ITN use, and were developed by modifying the respective items of a previous study instrument on diabetes mellitus [48], which had also been developed based on the same theory. Both sections had responses on Likert scales.

Motivation was assessed by asking them how good or bad they felt sleeping under an ITN was for their pregnancies, and how pleasant or unpleasant they felt it was. Self-efficacy on the other hand was assessed by asking of how easy or hard they felt it would be for them to sleep under an ITN every night; and how effectively or ineffectively they felt they could perform certain tasks related to ITN use and ITN care. Respondents’ use of ITN was categorized based on the number of days per week in which they slept under an ITN. Those who slept under an ITN for at least 3 days every week were categorized as ‘ITN users’, while those who slept under an ITN less frequently or not at all, were categorized as ‘ITN non-users’.

Pre-testing of the questionnaire performed on a sample of 190 respondents revealed Cronbach’s alpha of 0.87 for the section on motivation and 0.77 for the section on self-efficacy. For the test of reliability, 50 respondents out of the initial 190, were made to complete the questionnaire again after 2 weeks, and the Cohen’s Kappa scores for each of all the 23 items from the knowledge, motivation, and self-efficacy sections, were above 0.7, except one item in the knowledge section. Due to the low literacy rates, interviews by trained enumerators were used to obtain the information from the respondents.

### Statistical analysis and ethical consideration

The data obtained was analysed using IBM Statistical Package for Social Sciences (SPSS) version 22. Descriptive statistics with frequency and percentage were used to present the respondents’ responses. Bivariate analyses were performed using Chi squared test, to determine the association between ITN use and the factors studied. For this purpose, the respondents’ income levels were categorized into three: those with no income; those with income below the Nigerian minimum income wage (N18,000) and those with income up to, or above the minimum wage. The first sub-section for motivation was re-coded into three levels: ‘bad’ (comprising ‘very bad’ and ‘somewhat bad’); ‘neither good nor bad’, and ‘good’ (comprising of ‘somewhat good’ and ‘very good’). The second sub-section was also re-coded into three categories: ‘unpleasant’ (comprising ‘very unpleasant’ and ‘somewhat unpleasant’), ‘neither unpleasant nor pleasant’, and ‘pleasant’ (comprising ‘somewhat pleasant’ and ‘very pleasant’); while the third sub-section was re-coded into two categories: ‘untrue’ (comprising ‘very untrue’, ‘mostly untrue’, and ‘untrue’) and ‘true’ (comprising ‘true’, ‘mostly true’ and ‘very true’). For self-efficacy, both sub-sections were re-categorized into two thus: hard (comprising very hard and hard) and easy (comprising easy and very easy); and ineffectively (comprising very ineffectively and ineffectively) and effectively (comprising effectively and very effectively).

Multivariate logistic regression analysis was then performed on 18 variables, all of which had had a significance value of at least 0.25 or had shown very strong association with ITN-use, in previous studies. Following this, four variables were dropped, which did not significantly contribute to the model, leaving 14 variables, which were then analysed using the ‘ENTER’ method to obtain the final model.

Permission to conduct the study, as well as ethical clearance, was obtained from the Ethics Committee of the State Specialist Hospital (SSH/GEN/64/Vol.1) and Ethics Committee for Research Involving Human Subjects of the Universiti Putra Malaysia (UPM) (UPM/TNCPI/RMC/1.4.18.2). Informed consent was also obtained from the respondents after they had been taken through the respondent information sheet.

## Results

A total of 380 respondents were recruited from 30th January, 2017, to the 13th March, 2017. Their socio-demographic and maternal characteristics are presented in Table 1. Their ages were not normally distributed, with median (IQR) age of 25 (8) years; the median of which was used as the cut-off to dichotomize respondents into two age levels. The predominant ethnicity was Kanuri (35.8%), and over a half (58.9%) had at least a primary school education. Around a third (31.1%) of them were Internally Displaced Persons (IDP) from other local government areas of Borno State. Most of the respondents were unemployed (55.5%), and multiparous (64.5%).

**Table 1:** Respondents’ characteristics

Factor	Frequency (n)	Percentage (%)
Age		
Less than 25	33	(8.7)
25 years and above	347	(91.3)
Total	380	(100.0)
Age range	15–45	
Ethnicity		
Kanuri	136	(35.8)
Hausa	59	(15.5)
Babur	33	(8.7)
Fulani	39	(10.3)
Others	113	(29.7)
Total	380	(100.0)
Education		
None	156	(41.1)
Primary	67	(17.6)
Secondary	108	(28.4)
Tertiary	49	(12.9)
Total	380	(100.0)
Occupation status		
Employed	172	(45.3)
Not employed	208	(54.7)
Total	380	(100.0)
Income level		
None	211	(55.5)
Below minimum wage	140	(36.8)
At and above minimum wage	29	(7.6)
Total	380	(100.0)
Type of residence		
Permanent resident	279	(73.4)
Internally displaced	101	(26.6)
Total	380	(100.0)
Parity		
Nullipara	55	(14.5)
Primipara	80	(21.1)
Multipara	245	(64.5)
Total	380	(100.0)
Previous miscarriage		
Yes	75	(19.7)
No	305	(80.3)
Total	380	(100.0)
Previous miscarriage		
Yes	103	(27.1)
No	277	(72.9)
Total	380	(100.0)

Eighty percent of them were aware of insecticide-treated nets (ITNs), as shown in Table 2. A half of them (50.5%) believed that the chemicals on the ITNs could be dangerous to those sleeping under them. Less than a third (26.1%) knew that ITNs should be washed after 3–4 months, while 61.3% knew that ITNs should be dried under the shade after washing.

**Table 2:** Respondents’ knowledge of ITN

Insecticide-treated net (ITN) use and care	Response					
	Correct		Incorrect		I don’t know	
	n	(%)	n	(%)	n	(%)
Are you aware of ITN?	305	(80.3)	48	(12.6)	27	(7.1)
ITNs are used to keep mosquitoes away	320	(84.2)	43	(11.3)	17	(4.5)
ITNs are used to keep rats away	200	(52.6)	153	(40.3)	27	(7.1)
ITNs are more effective compared to plain nets	225	(59.2)	110	(28.9)	45	(11.8)
The chemicals on an ITN can be dangerous to one who sleeps under it	133	(35.0)	192	(50.5)	55	(14.5)
ITNs should be washed after every 1 month	110	(28.9)	225	(59.2)	45	(11.8)
ITNs nets should be washed after every 3–4 months	99	(26.1)	198	(52.1)	83	(21.8)
ITNs should be washed after every 6 months	248	(65.3)	36	(9.5)	96	(25.3)
ITNs should be washed with water and ordinary soap only	206	(54.2)	129	(33.9)	45	(11.8)
ITNs should be washed with water and detergent	135	(35.5)	205	(53.9)	40	(10.5)
ITNs should be dried under the shade after washing	233	(61.3)	112	(29.5)	35	(9.2)
ITNs should be dried under the sun after washing	157	(41.3)	184	(48.4)	39	(10.3)

Respondents’ motivation for ITN use is presented in Table 3. Less than one percent of them believed that sleeping under an ITN during the period of their pregnancies was very bad for their health, while 1.1% felt that sleeping under an ITN was very unpleasant. Thirty percent of them stated that it was very true that their significant others thought they should sleep under an ITN during their pregnancies.

**Table 3:** Respondents’ motivation for ITN use

Statement	Response					
For the remaining duration of your pregnancy, how good or bad would it be for your health	Very bad	Somewhat bad	Neither bad nor good	Somewhat good	very good	
To sleep under an ITN?	3 (0.8)	21 (5.5)	28 (7.4)	109 (28.7)	219 (57.6)	
To sleep more frequently under an ITN?	24 (6.3)	14 (3.7)	58 (15.3)	139 (36.6)	145 (38.2)	
For the remaining duration of your pregnancy, how pleasant or unpleasant would it be for you	Very unpleasant	Somewhat unpleasant	Neither unpleasant nor pleasant	Somewhat pleasant	Very pleasant	
To sleep under an ITN?	4 (1.1)	8 (2.1)	34 (36.3)	138 (36.3)	196 (51.6)	
To sleep more frequently under an ITN?	21 (5.5)	16 (4.2)	60 (15.8)	147 (38.7)	136 (35.8)	
How true or untrue is it, that most people who are important to you think you should	Very untrue	Mostly untrue	Untrue	True	Mostly true	Very true
Sleep under an ITN?	6 (1.6)	7 (1.8)	21 (5.5)	131 (34.5)	103 (27.1)	112 (29.5)
Sleep more frequently under an ITN?	3 (0.8)	7 (1.8)	70 (18.4)	124 (32.6)	86 (22.6)	90 (23.7)

The respondents’ self-efficacies for using ITN use are presented in Table 4. Over a half of them felt it would be very easy for them to sleep under an ITN every night during their pregnancies. A half (51.3%) believed they could hang an ITN very effectively, but less than a half of them (42.9%) felt they could very effectively persuade others to support their sleeping under an ITN.

**Table 4:** Respondents’ self-efficacy for ITN use

Statement	Response			
Right now, how easy or hard would it be for you to…	Very hard	Hard	Easy	Very easy
Sleep under an ITN every night?	5 (1.3)	9 (2.4)	152 (40.0)	214 (56.3)
Right now, how effectively or ineffectively could you…	Very ineffectively	Ineffectively	Effectively	Very effectively
Properly hang your ITN?	8 (2.1)	26 (6.8)	151 (39.7)	195 (51.3)
Check for and repair holes and rifts in your ITN?	9 (2.4)	45 (11.8)	162 (42.6)	164 (43.2)
Sleep more frequently under an ITN?	8 (2.1)	74 (19.5)	149 (39.2)	149 (39.2)
Persuade others to support your sleeping under an ITN?	16 (4.2)	61 (16.1)	140 (36.8)	163 (42.9)

Thirty-five percent (35.0%) of the respondents were ITN users. Table 5 shows the bivariate association between respondents’ characteristics and ITN use. Having no history of previous miscarriage was the only factor significantly associated with ITN use. Association between ITN knowledge and its use is presented in Table 6. Being aware of ITN, knowing that ITNs should be washed after 3–4 months, and that they should be washed with only water and ordinary soap, and not with detergents, all showed significant association with ITN use.

**Table 5:** Association between respondents’ characteristics and ITN use

Variables	ITN use status				*χ* ^*2*^	*df*	*p*
	Non user freq. (%) n = 247		User freq. (%) n = 133				
Age group					1.465	1	0.226
Less than 25	103	(10.5)	47	(5.3)			
25 years and above	144	(89.5)	86	(94.7)			
Ethnicity					0.099	1	0.753
Kanuri	87	(35.2)	49	(36.8)			
Others	160	(64.8)	84	(63.2)			
Type of residence					0.161	1	0.688
Permanent resident	183	(74.1)	96	(72.2)			
IDP	64	(25.9)	37	(27.8)			
Education level					0.840	3	0.840
None	98	(39.7)	58	(43.6)			
Primary	46	(18.6)	21	(15.8)			
Secondary	70	(28.3)	38	(28.6)			
Tertiary	33	(13.4)	16	(12.0)			
Occupational status					1.268	1	0.260
Not employed	143	(57.9)	69	(51.9)			
Employed	104	(42.1)	64	(48.1)			
Income level					1.102	2	0.576
None	142	(57.5)	69	(51.9)			
Below minimum wage	87	(35.2)	53	(39.8)			
At and above minimum wage	18	(7.3)	11	(8.3)			
Parity					3.666	2	0.160
Nullipara	42	(17.0)	13	(9.8)			
Primipara	50	(20.2)	30	(22.6)			
Multipara	155	(62.8)	90	(67.7)			
Total	247	(100.0)	133	(100.0)			
Previous preterm delivery					2.414	1	0.120
Yes	43	(17.4)	32	(24.1)			
No	204	(82.6)	101	(75.9)			
Previous miscarriage					9.818	1	0.002*
Yes	54	(21.9)	49	(36.8)			
No	193	(78.1)	84	(63.2)			

* Significant *p* < 0.05

**Table 6:** Association between ITN knowledge and ITN use

Variables	ITN use status				*χ* ^*2*^	*df*	*p*
	Non user freq. (%) n = 247		User freq. (%) n = 133				
Aware of ITN					9.609	2	0.008*
I don’t know	24	(9.7)	3	(2.3)			
Correct	188	(76.1)	117	(88.0)			
Incorrect	35	(14.2)	13	(9.8)			
Total	247	(100.0)	133	(100.0)			
ITN to keep mosquitoes away					4.254	2	0.119
I don’t know	15	(6.1)	2	(1.5)			
Correct	204	(82.6)	116	(87.2)			
Incorrect	28	(11.3)	15	(11.3)			
Total	247	(100.0)	133	(100.0)			
ITN to keep rats away					1.109	2	0.574
I don’t know	19	(7.7)	8	(6.0)			
Correct	95	(38.5)	58	(43.6)			
Incorrect	133	(53.8)	67	(50.4)			
Total	247	(100.0)	133	(100.0)			
ITN more effective than plain nets					0.483	2	0.786
I don’t know	25	(10.1)	11	(8.3)			
Correct	56	(22.7)	33	(24.8)			
Incorrect	166	(67.2)	89	(66.9)			
Total	247	(100.0)	133	(100.0)			
The chemicals on ITNs are dangerous					1.716	2	0.424
I don’t know	40	(16.2)	15	(11.3)			
Correct	123	(49.8)	69	(51.9)			
Incorrect	84	(34.0)	49	(36.8)			
Total	247	(100.0)	133	(100.0)			
ITNs should be washed after 1 month					1.562	2	0.458
I don’t know	33	(13.4)	12	(9.0)			
Correct	144	(58.3)	81	(60.9)			
Incorrect	70	(28.3)	40	(30.1)			
Total	247	(100.0)	133	(100.0)			
Should be washed after 3–4 months					6.508	2	0.039*
I don’t know	56	(22.7)	27	(20.3)			
Correct	137	(55.5)	61	(45.9)			
Incorrect	54	(21.9)	45	(33.8)			
Total	247	(100.0)	133	(100.0)			
Should be washed after 6 months					3.382	2	0.184
I don’t know	64	(25.9)	32	(24.1)			
Correct	28	(11.3)	8	(6.0)			
Incorrect	155	(62.8)	93	(69.9)			
Total	247	(100.0)	133	(100.0)			
Should be washed with water and soap only					10.526	2	0.005*
I don’t know	28	(11.3)	17	(12.8)			
Correct	98	(39.7)	31	(23.3)			
Incorrect	121	(49.0)	85	(63.9)			
Total	247	(100.0)	133	(100.0)			
Should be washed with water and detergent					12.163	2	0.002*
I don’t know	18	(7.3)	22	(40)			
Correct	147	(59.5)	58	(205)			
Incorrect	82	(33.2)	53	(39.8)			
Total	247	(100.0)	133	(100.0)			
ITNs should be dried under the shade					2.638	2	0.261
I don’t know	20	(8.1)	15	(11.3)			
Correct	79	(32.0)	33	(24.8)			
Incorrect	148	(59.9)	85	(63.9)			
Total	247	(100.0)	133	(100.0)			
ITNs should be dried under the sun					4.652	2	0.098
I don’t know	20	(8.1)	19	(14.3)			
Correct	127	(51.4)	57	(42.9)			
Incorrect	100	(40.5)	57	(42.9)			
Total	247	(100.0)	133	(100.0)			

* Significant *p* < 0.05

Having significant others who thought they should sleep under an ITN, and to do so more frequently, was associated with ITN use as shown in Table 7. Also as presented in Table 8, the ability to effectively hang a net properly, effectively check for and repair holes and rifts in the net, effectively sleep more frequently under an ITN, and the ability to effectively persuade others to sleep under one, were all significantly associated with ITN use.

**Table 7:** Association between motivation and ITN use

Questions/statements	Group				*χ* ^*2*^	*df*	*p*
	Non user freq. (%) n = 247		User freq. (%) n = 133				
For you to sleep under an ITN is					0.385	2	0.825
Neither bad nor good	17	(6.9)	7	(5.3)			
Good	18	(7.30)	10	(7.5)			
Bad	212	(85.8)	116	(87.2)			
Total	247	(100.0)	133	(100.0)			
For you to sleep more frequently under an ITN is					3.555	2	0.169
Neither bad nor good	28	(11.3)	10	(7.5)			
Good	42	(17.0)	16	(12.0)			
Bad	177	(71.7)	107	(80.5)			
Total	247	(100.0)	133	(100.0)			
For you to sleep under an ITN is					0.016	2	0.992
Neither unpleasant nor pleasant	8	(3.2)	4	(3.0)			
Pleasant	22	(8.9)	12	(9.0)			
Unpleasant	217	(87.9)	117	(88.0)			
Total	247	(100.0)	133	(100.0)			
For you to sleep more frequently under an ITN is					1.009	2	0.604
Neither unpleasant nor pleasant	25	(10.01)	12	(9.0)			
Pleasant	42	(17.0)	18	(13.5)			
Unpleasant	180	(72.9)	103	(77.4)			
Total	247	(100.0)	133	(100.0)			
Most people who are important to you think you should sleep under an ITN					6.760	1	0.009*
Untrue	29	(11.7)	5	(3.8)			
True	218	(88.3)	128	(96.2)			
Total	247	(100.0)	133	(100.0)			
Most people who are important to you think you should sleep more frequently under an ITN					10.022	1	0.002*
Untrue	64	(25.9)	16	(12.0)			
True	183	(74.1)	117	(88.0)			
Total	247	(100.0)	133	(100.0)			

* Significant *p* < 0.05

**Table 8:** Association between self-efficacy and ITN use

Variables	Group				*χ* ^*2*^	*df*	*p*
	Non user Freq. (%) n = 247		User Freq. (%) n = 133				
Right now, how easy or hard would it be for you to…							
Sleep under an ITN every night?					0.39	1	0.530
Hard	8	(3.2)	6	(94.5)			
Easy	239	(96.8)	127	(95.5)			
Total	247	(100.0)	133	(100.0)			
Properly hang your ITN?					4.94	1	0.026*
Ineffectively	28	(11.3)	6	(4.5)			
Effectively	219	(88.7)	127	(95.5)			
Total	283	(100.0)	97	(100.0)			
Check for and repair holes and rifts in your ITN?					4.52	1	0.034*
Ineffectively	42	(17.0)	12	(9.0)			
Effectively	205	(83.0)	121	(91.0)			
Total	283	(100.0)	97	(100.0)			
Sleep more frequently under an ITN?					7.83	1	0.005*
Ineffectively	64	(25.9)	18	(13.5)			
Effectively	183	(74.1)	115	(86.5)			
Total	283	(100.0)	97	(100.0)			
Persuade others to support your sleeping under an ITN?					10.22	1	0.001*
Ineffectively	62	(25.1)	15	(11.3)			
Effectively	185	(74.9)	118	(88.7)			
Total	283	(100.0)	97	(100.0)			

* Significant *p* < 0.05

For the multivariate logistic regression, the model fitted the sample, evidenced by a Hosmer–Lemeshow significance value of 0.355. The Negelkerke’s R square also showed that the model explained about 25.1% of the variation in ITN use. The predictors of ITN use are presented in Table 9. Those with no history of previous miscarriage were twice more likely to be ITN users compared to those who had a previous miscarriage. Those who knew that ITNs were not to be washed after 1 month were over thrice more likely to be ITN users compared to those who were not sure. Contrastingly, those who correctly stated that detergents should not be used, as well as those who wrongly stated that detergents could be used to wash ITNs were less likely to be ITN users compared to those who did not know whether or not it could be used. Those whose significant others thought they should sleep under an ITN were thrice more likely to be ITN users compared to those whose significant others thought otherwise. Also, those who could effectively persuade others to support their sleeping under an ITN were twice more likely to be ITN users compared to those who could not effectively do so.

**Table 9:** Predictors of ITN use

Factors	B	SE	Wald	*df*	*p*	Adjusted OR	95% CI
Age							
Less than 25						1	
25 years and above	0.25	0.26	0.94	1	0.333	1.28	0.78–2.12
Ethnicity							
Kanuri						1	
Others	− 0.14	0.26	0.31	1	0.58	0.87	0.52–1.43
Miscarriage							
No						1	
Yes	0.87	0.27	10.48	1	0.001*	2.38	1.41–4.03
Aware of ITN							
I don’t know						1	
Correct	1.73	0.70	6.10	1	0.014*	5.66	1.43–22.42
Incorrect	1.64	0.79	4.28	1	0.038*	5.18	1.09–24.55
ITNs are for keeping mosquitoes away							
I don’t know						1	
Correct	1.40	0.89	2.47	1	0.116	4.06	0.71–23.38
Incorrect	1.03	0.96	1.15	1	0.284	2.81	0.42–18.59
ITNs should be washed after every 1 month							
I don’t know						1	
Correct	1.28	0.57	5.03	1	0.025*	3.60	1.18–11.06
Incorrect	1.06	0.61	3.04	1	0.081	2.89	0.88–9.50
ITNs should be washed with water and ordinary soap only							
I don’t know						1	
Correct	− 0.14	0.53	0.07	1	0.791	0.87	0.31–2.45
Incorrect	0.53	0.50	1.12	1	0.290	1.69	0.64–4.48
ITNs should be washed with water and detergent							
I don’t know						1	
Correct	− 2.08	0.60	12.04	1	0.001*	0.13	0.04–0.40
Incorrect	− 1.69	0.60	7.95	1	0.005*	0.18	0.06–0.60
To sleep under an ITN is							
Neither unpleasant nor pleasant						1	
Pleasant	0.45	0.96	0.22	1	0.637	1.57	0.24–10.28
Unpleasant	0.41	0.49	0.70	1	0.403	0.66	0.25–1.74
To sleep more frequently under an ITN is							
Neither unpleasant nor pleasant						1	
Pleasant	0.12	0.56	0.05	1	0.832	1.13	0.38–3.36
Unpleasant	0.82	0.44	3.47	1	0.062	0.44	0.19–1.04
Most people who are important to you think you should sleep under an ITN							
Untrue						1	
True	0.89	0.62	2.02	1	0.155	2.43	0.72–8.23
Most people who are important to you think you should sleep more frequently under an ITN							
Untrue						1	
True	1.12	0.42	7.15	1	0.007*	3.06	1.35–6.96
To sleep under an ITN every night is							
Hard						1	
Easy	− 0.75	0.69	1.18	1	0.277	0.47	0.12–1.83
You can persuade others to support your sleeping under an ITN							
Ineffectively						1	
Effectively	0.86	0.37	5.31	1	0.021*	2.37	1.14–4.94

* Significant *p* < 0.05

## Discussion

A higher proportion of women in this study had some level of formal education compared to the general female population of Borno state in 2013 (58.9% against 27.6%) [26]. A prior study of malaria in pregnancy at the same study area had also revealed a lower level of education (43.5%) [10]. Conflicting results have been reported on the association between education level and ITN use. While some studies showed a higher use of ITN with increasing level of education and income [49, 50], others showed the opposite [51, 52]. However, the findings of this study appear in line with the former, as a higher ITN use was also reported among its respondents. There was however no significant association between age, level of education, ethnicity, occupation and ITN use, similar to a previous study in the same region in Nigeria [9]. The lower use of ITNs among those with history of previous abortions could buttress earlier reports of malaria being a huge contributor to abortions in malaria endemic areas [53], since those women were at higher risks of being bitten by malaria infected mosquitoes, which increased their chances of having an abortion.

A relatively higher proportion of women in this study were aware of ITNs (80.3%) compared to a previous one in south-western Nigeria, where 77.6% of them were aware of ITNs [54]. Compared to a previous study [47], even though a little more in this study correctly mentioned that ITNs were used to prevent mosquito bites (84.2% versus 82.4%), more also wrongly believed that it was used to keep rats away (40.3% versus 27.1%). A lower proportion of women in this study were aware of the effectiveness and safety of ITNs, compared to a previous study in a rural community in the south-west, where only 6.3% believed that plain nets were better than ITNs, while only 0.3% believed that using ITNs in pregnancy could cause a miscarriage [47]. As in this study, it had previously been reported that awareness of ITN was associated with ITN use [55], which is only logical, as those using ITNs should be aware of it. Similarly, a prior study in Ethiopia had shown that those who had received some information about malaria were more likely to use ITNs compared to those who had not [56]. However, in contrast to a previous study in Nigeria where not holding any misconceptions about ITN was a predictor of using it [57], this study showed no difference in ITN use between those who believed it could be harmful and those who believed it was safe.

Most of the respondents demonstrated positive attitudes towards ITN use, as over 70% of them felt that it was pleasant to use, and also believed it was also good for their pregnancies. When the level of ITN use in this study is compared to findings from an earlier study in which the respondents had a higher awareness of ITN (82.4%) but lower positive attitudes towards its use (20.9%) [58], it could be inferred that motivation likely plays a bigger role compared to knowledge, in influencing ITN use. The main system of ITN delivery to pregnant women in sub-Saharan Africa has been through free distribution at antenatal clinics [59], and as such, it was as expected that only a very small proportion of them felt that sleeping under it was either very bad or somewhat bad to their health, as it is unlikely for them to assume that health workers would give them any harmful material to use. Significant correlation has been reported between husband’s permission and antenatal care access among women in northern Nigeria [60]. In another study in north-eastern Nigeria, 25.7%, 15.5% and 17.1%, respectively, stated problems of obtaining permission from their spouses, parents/guardians and religious/cultural leaders as their reason for not going for antenatal care [61]. Considering this prevailing culture in the region, a husband who would allow his apparently healthy wife to go for antenatal care visits is likely to be a very supportive one, which could explain the high level of social support for ITN use reported in this study. The thought of sleeping under an ITN being pleasant or not, was not associated with ITN use, implying that both ITN users and non-users equally felt that ITNs were either pleasant or not. Despite unpleasant feelings like heat [52, 62] being reported as the reason for non-use of ITN in other studies, it can be seen that having supportive people around plays a significant role in overcoming these obstacles.

Most of the participants found sleeping under an ITN, or taking care of it as either easy or very easy, and most said they could do them either effectively or very effectively. Even though the ability to hang a net had been reported to predict its use among pregnant women in Congo [34], it did not predict ITN use in this study probably because they were likely to have their nets hung for them by others, which should not be surprising, considering the fact that 78.9% of them had reported that their significant others believed in ITN use. Household decision had been earlier identified as a significant determinant of ITN use [30], similar to this study where those whose significant others thought they should sleep under an ITN were more likely to do so. Ability to effectively persuade their significant others to support their choice of sleeping under an ITN was the only item from the self-efficacy section that significantly predicted ITN use. This could comprise the ability to persuade others to hang the nets for them, and/or ability to positively influence household decisions towards supporting ITN use by them.

In this study, the proportion of those sleeping under an ITN during their current pregnancy (42.63%), was higher than overall reports from Borno state, where only 13.8% were sleeping under ITNs [26]. Antenatal care attendees of a tertiary centre in the same city, had previously in 2009 shown lower levels of ITN use (2.3%) [7], indicating a probable increase in general public awareness about malaria, considering the time difference between the two studies. It could also be because the tertiary health centres are more likely to be attended by women of higher social status, compared to those attending the secondary-level health facility, and lower level of ITN use has been reported among the more educated and the wealthier [4, 52].

The strengths of this study include the adequate number of respondents recruited, as this was over twice the minimum calculated sample size. The similarity of age range in this study with that of the Demographic and Health Survey of 2013 (15–45 versus 15–49) [26], and similar distribution of parities (nulliparous—14.5% against 31.5%, primiparous—21.1% against 14.5%, multiparous—41.1% against 39% and grand multiparous—23.4% against 15%) with a previous study in a tertiary hospital in Maiduguri [7], could allow comparability of findings. Another strength of this study was the multivariate analysis performed which controls for confounding factors.

Among the limitations of the study were the inability to determine the exact temporal relationship between the variables since it was a cross-sectional study. Also, collapsing multi-level variables of the motivation and self-efficacy scales into dichotomous or three-levels must have resulted in loss of some information. This was however to enable the conduct of the multivariate analysis.

## Conclusion

A large proportion of respondents in this study were not sleeping under an ITN. The results suggest that interventions aimed at increasing their awareness of ITN, as well as self-efficacy have the potentials of increasing compliance with ITNs. It is also recommended that health promotion programmes also focus on motivating the important family members, like husbands to support their spouses to use ITNs. Counselling on ITN use, should also be incorporated into post-abortal care. The model only explained 25% of ITN use among the respondents, suggesting that there may be several other factors influencing this practice which were not studied. Qualitative studies are recommended to further explore these factors.
